# Cotinine Enhances Fear Extinction and Astrocyte Survival by Mechanisms Involving the Nicotinic Acetylcholine Receptors Signaling

**DOI:** 10.3389/fphar.2020.00303

**Published:** 2020-04-02

**Authors:** Patricia Oliveros-Matus, Nelson Perez-Urrutia, Nathalie Alvarez-Ricartes, Florencia Echeverria, George E. Barreto, James Elliott, Alexandre Iarkov, Valentina Echeverria

**Affiliations:** ^1^Laboratorio de Neurobiología, Facultad de Ciencias de la Salud, Universidad San Sebastián, Concepción, Chile; ^2^Department of Biological Sciences, University of Limerick, Limerick, Ireland; ^3^Health Research Institute, University of Limerick, Limerick, Ireland; ^4^Northern Sydney Local Health District, The Kolling Research Institute and Faculty of Health Sciences, The University of Sydney, St. Leonards, NSW, Australia; ^5^Department of Physical Therapy and Human Movement Sciences, Feinberg School of Medicine, Northwestern University, Chicago, IL, United States; ^6^Research and Development Department, Bay Pines VA Healthcare System, Bay Pines, FL, United States

**Keywords:** fear conditioning, bone morphogenetic proteins, prefrontal cortex, cholinergic receptors, MLA

## Abstract

Fear memory extinction (FE) is an important therapeutic goal for Posttraumatic stress disorder (PTSD). Cotinine facilitates FE in rodents, in part due to its inhibitory effect on the amygdala by the glutamatergic projections from the medial prefrontal cortex (mPFC). The cellular and behavioral effects of infusing cotinine into the mPFC on FE, astroglia survival, and the expression of bone morphogenetic proteins (BMP) 2 and 8, were assessed in C57BL/6 conditioned male mice. The role of the α4β2- and α7 nicotinic acetylcholine receptors (nAChRs) on cotinine’s actions were also investigated. Cotinine infused into the mPFC enhanced contextual FE and decreased BMP8 expression by a mechanism dependent on the α7nAChRs. In addition, cotinine increased BMP2 expression and prevented the loss of GFAP + astrocytes in a form independent on the α7nAChRs but dependent on the α4β2 nAChRs. This evidence suggests that cotinine exerts its effect on FE by modulating nAChRs signaling in the brain.

## Introduction

Posttraumatic stress disorder (PTSD) represents a consequential set of reactions to a traumatic event(s) perceived as threatening life and resulting in the person experiencing feelings of intense helplessness and horror. Management strategies for PTSD include therapies to facilitate fear memory extinction (FE), which have shown to inhibit fear responses (Rutt et al., 2018), but there is a paucity of evidence to support long-term pharmacological and psychotherapeutic treatment options (Wessa and Flor, 2007). Furthermore, even if FE is achieved with treatment, between 60 and 72% of patients with PTSD re-experience a return of their symptoms, or suffer for many years of them raising questions around the mechanisms underlying effective short- and long-term FE (Steenkamp et al., 2015).

Several mechanistic studies suggest the medial prefrontal cortex (mPFC) plays a role in the consolidation of FE learning (Quirk et al., 2000). Previous studies revealed that cotinine, is a positive allosteric modulator (PAM) type II of the α7 nicotinic acetylcholine receptors (nAChRs), enhanced contextual FE, by mechanisms involving the activation of the extracellular-signal regulated kinases (ERKs) (Zeitlin et al., 2012) and calcineurin A (CaA) (Alvarez-Ricartes et al., 2018b) in the hippocampus (HIP) of mice (Mendoza et al., 2018). In addition, orally administered cotinine, prevented the loss of synaptic density in the PFC and HIP of chronically stressed male mice (Grizzell et al., 2014). PAMs type II of the nAChRs bind to allosteric sites inducing a positive effect on receptor activity, by stabilizing its active conformation and preventing its desensitization. This concept describes very well the effect of cotinine that is a weak agonist of the α7nAChR to the orthostatic site, but positively modulates the activity of the human α7 receptors, increasing the amplitude of the currents induced by nicotine and acetylcholine *in Xenopus* oocyte expressing the human receptor (Terry et al., 2015). Altogether, we proposed that cotinine is a positive allosteric modulator of the receptor, though, we cannot discard that cotinine may affect other effectors that indirectly act over the nAChRs to modulate its activity. At our knowledge the model of *Xenopus* Oocytes used by Terry and others is one of the best models to characterize the modulation of the nicotinic receptors by new drugs to minimize the risk of being affecting other human effectors that will not be present in the oocyte. However, structure-based docking studies investigating the binding of cotinine to the α7 nAChRs, combined to cell based binding and activity studies to test the ″docking hits″ using mutagenesis, it could be used to determine the predicted direct interaction of cotinine and the nicotinic receptors subunits as well as the nature of the allosteric modulation involved. We have found that Cotinine alters the mRNA expression of BMP2 and BMP8 in the brain of restrained mice (unpublished observation). Because BMPs control the expression of stress factors, we hypothesized that their expression could also be affected by cotinine during fear extinction by mechanisms dependent on the nAChRs. The neurobiological mechanisms underlying the psychological and physical consequences of trauma affecting FE appear to also involve changes in the HPA axis components affecting astrogenesis (Perez-Urrutia et al., 2017). In this respect, the bone morphogenetic proteins (BMPs), members of the transforming growth factor (TGFβ) family, broadly expressed in the brain, play an important function in the CNS regulating astrogenesis (Imura et al., 2008) and neurogenesis during development (Shaked et al., 2008; Morikawa et al., 2016) and adulthood (Colak et al., 2008), and perhaps their function can be affected in the brain by PTSD.

The primary purpose of this study was to determine the cellular and behavioral effects of infusing cotinine into the PFC on FE and the expression of bone morphogenetic proteins (BMP) 2 and 8, in C57BL/6 male mice. A secondary purpose is to identify the role of the α4β2- and α7nAChRs on cotinine’s behavioral, cellular and molecular actions. This investigation revealed that the infusion of cotinine into the mPFC enhanced contextual FE, glial fibrillary acidic protein (GFAP) + astrocytes survival, and the BMP2/8 expression in the brain of conditioned mice by a mechanism dependent on the nAChRs.

## Materials and Methods

### Drugs

Cotinine (5S-1-methyl-5-(3-pyridyl) pyrrolidine-2-ona) was obtained from Sigma-Aldrich (Saint Louis, MO, United States). α4β2nAChR inhibitor dihydro-beta-erythroidine (DHβE) and the α7nAChR inhibitor, methyllycaconitine (MLA) were obtained from Tocris bioscience (Bristol, United Kingdom).

### Animals

Male C57BL/6 mice, 3–4 months of age and 30–35 g of weight, were obtained from the University of Chile (Santiago, Chile) and maintained on a 12 h/12h light-dark cycle with *ad libitum* access to food and water. Mice were maintained grouped (2–4 mice by a cage) in a controlled environment with average temperatures between 23–25°C and 50–70% humidity. The protocols were designed to minimize animal suffering and reduce the number of animals used. Animal studies were performed in compliance with the ARRIVE guidelines (Kilkenny et al., 2010) and after the approval of the Institutional animal care and use committees of the University of San Sebastián, Chile according to the Guide of Animal care and use of laboratory animals of the National Institute of Health (NIH publication 80-23/96). The authors declare that every effort was made to reduce the number of mice used and their level of disconfort and suffering.

### Experimental Design

**FIGURE 1:**
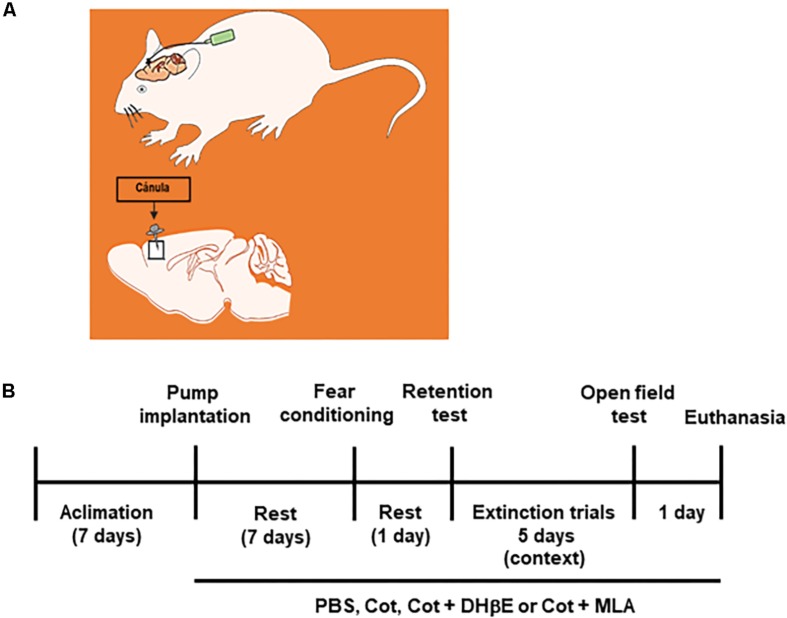
Experimental design. **(A)** the drawing represents the intracerebral infusion of treatments by osmotic pumps by continues delivery into the frontal cortex of mice. **(B)** Time line of treatments, behavioral tests and final experimental procedures in the study.

This study investigated the effect of cotinine infused directly into the mPFC on depressive-like behavior, fear consolidation and extinction as well as the expression of BMPs in the PFC and HIP of mice subjected or not to fear conditioning (FC) and FE (Figure 1).

### Drug Treatments

Three-month-old mice (*n* = 8–13/condition) were weighed and randomly assigned to treatment groups. Mice received continuous infusions with vehicle or drugs delivered chronically using ALZET^®^ Osmotic Pumps, infusion pumps for continuous dosing of freely moving mice, for 2 weeks, starting 1 week before FC until euthanasia. Mice received treatments with phosphate buffer saline (PBS, pH 7.4), Cotinine (5 mg/ml) dissolved in PBS (Cot 5), DHβE (2.14 μg/ml in PBS, 100 μl, 13 μg/day), MLA (0.24 mg/ml in PBS, 100 μl, 3 μg/day, 3.3 nmoles), Cot 5 plus DHβE (2.14 mg/ml in PBS, 100 μl, 13 μg/day), and Cot 5 plus MLA (0.24 mg/ml in PBS, 100 μl, 3 μg/day). The dose of cotinine was based on the dose of IN cotinine that promoted FE in C57BL/6 mice (Alvarez-Ricartes et al., 2018a). The dose of DHβE and MLA was chosen based on previous studies (Laurier et al., 1994; Yeomans and Baptista, 1997; Panagis et al., 2000; Laviolette and van der Kooy, 2003). The doses of DHβE (10–300 μg, equivalent to 28–840 nmol) (Yeomans and Baptista, 1997) and MLA (2, 6, 18 μg totales) have been shown to be effective inducing behavioral changes following intra-cranial administration (Panagis et al., 2000). We choose the lower dose range for our experiments, considering that a continue infusion of drugs will require lower doses than one-time intracranial administrations.

### Infusion of Drugs Into the Prefrontal Cortex

The infusion of drugs into the mPFC was performed using osmotic pumps as previously described (Besson et al., 2007). First, mice were subjected to behavioral analysis and then subjected to a stereotaxic surgery under anesthesia, with a mix including Ketamine, xylazine, and acepromazin (80, 8 and 1 mg/kg), to implant a cannula in the medial prefrontal cortex (AP: + 2.0 mm; L 1.5 mm, V 3.5 mm, angle 30°) attached to a Alzet^®^ osmotic pump (Alzet, Cupertino, CA, United States) filled with 100 μl of PBS alone (Vehicle) or a drug solution in PBS. The rate of delivery of drugs of the device was 0.25 μl/h and was kept for 14 days until the end of experiments. One week after surgery mice underwent FC and FE.

### Behavioral Analysis

One week following pump implantation, mice were conditioned and 24 h later tested in the fear retention test. On the next day, mice were subjected to daily FE trials for 5 days. Following FE trials, mice were tested for locomotor function using the open field test.

#### Fear Conditioning

Fear conditioning was performed as reported (Zeitlin et al., 2012). The conditioning chamber (33 cm × 20 cm × 22 cm) is surrounded by a sound-attenuating box that generates a background white noise (72 dB) and contains a camera at the top of the chamber connected to a computer. The conditioning chamber contains a speaker on one side and a 24 V light on the other, with a 36-bar insulated shock grid floor. Each mouse was placed in the chamber for 2 min before the onset of a tone lasting 30 s (2,800 Hz and 85 dB). In the last 2 s of the tone, mice received a foot shock of 1 mA, kept in the conditioning chamber for 2 min, and then returned to their home cages. The chamber was sanitized with 70% ethanol and dried after each trial. Freezing behavior was defined as the absence of all movement (except for movement secondary to breathing) and was assessed using Freezing behavior was measured using the Any-maze^®^ software (Stoelting, CO, United States).

#### Fear Retention and Extinction Tests

Fear retention and FE experiments were performed as previously described using the same cohorts of mice. To assess FR, mice underwent re-exposure to the conditioning chamber, in the absence of the unconditioned stimuli (foot-shock and auditory cues), for 3 min in daily extinction trials. The extinction trials persisted until the decrease in freezing behavior reached an asymptote.

#### Open Field Test

Open field is a reliable test evaluating the locomotor activity and exploratory behavior in mice (Walsh and Cummins, 1976). A period of pre-test habituation was carried out in which the mice were placed individually in the apparatus for 10 min on two consecutive days. The OF test device consisting of a square, open-top, arena (40 cm × 40 cm × 35 cm) that was virtually divided into two areas: peripheral and central zones. The mice were placed in the corner of the arena and allowed to roam freely for 25 min. The time (min), distance (m), and speed (m/sec) of the mice in the arena were recorded. The behavior of mice was documented using a camera located above the arena and analyzed with the software Any-Maze (Stoelting).

### Brain Tissue Preparation

For the protein studies, after euthanasia by cervical dislocation, the brains were removed, and the left hemispheres were dissected and quickly frozen for Western blot analyses. The right hemisphere was placed in 4% paraformaldehyde in PBS pH 7.4 at 4°C for 24 h, and then embedded in 2% agarose molds.

Brain regions of interest were located using the Paxinos Atlas (Paxinos and Franklin, 2001), and serial sections of 20 μm (*n* ≥ 3/mouse) were collected using the Vibratome Leica VT1000S and mounted on positively charged slides (Biocare Medical, Concord, CA, United States) for the immunohistochemistry (IHC) analysis.

### Western Blot Analysis

The protein analysis was performed using Western blot according to our previously published protocol (Alvarez-Ricartes et al., 2018b). The brain tissues were homogenized by sonication in lysis buffer with phosphatase and proteases inhibitors (Cell signaling technology, Danver, MA, United States). The homogenates were centrifuged, and aliquots of the supernatants used for the determination of protein concentration using the Biorad kit (Bio-Rad, Hercules, CA, United States). Brain extracts in SDS-denaturant loading buffer with equal amounts of protein were separated by electrophoresis in an SDS-PAGE (4–20%) gel and transferred to nitrocellulose membranes. After the transfer, membranes were blocked with TBS containing 0.1% of Tween 20 (TBST) and skim milk 10% (w/v) for 45 min and incubated with primary and secondary antibodies. The primary antibodies used included the rabbit polyclonal anti-BMP8 (United States Biological life sciences, Marblehead MA, United States, # 221074), rabbit polyclonal anti-BMP2 (Invitrogen PH131215), anti-Calcineurin A (Cell signaling technology) and mouse monoclonal anti-GFAP (Bio SB, BSB5567, Santa Barbara, CA, United States). For protein loading and transfer control, rabbit polyclonal antibody against β-actin (# 662102, Biolegend, San Diego, CA, United States) and rabbit monoclonal antibody against β-Tubulin (Cell signaling technology) were used. After incubation with antibodies, membranes were washed and analyzed for immunoreactive (IR) bands using the ECL imaging system and the NIH image J software (National Institute of Health, Bethesda, MA, United States).

### GFAP + Cells and BMP8 Immunohistochemical Analysis

The analysis of GFAP + cells and BMP8 IR was performed as previously described. Sagittal sections of the mPFC (AP: + 1.8 mm, ML: 0.0 mm, DV: −2.5 mm) and the HIP (Approx. Bregma −4.08 mm, interaural 4.92 mm) were collected in PBS and processed for GFAP and BMP8 IR.

Brain slices were immersed in xylene and decreasing the graduation of ethanol baths for hydration. Slices were then subjected to antigenic recovery in buffer citrate (pH = 6) in a pressurized saucepan (Biocare Medical, Walnut Creek, CA, United States) for 30 min, and incubated in a 3% hydrogen peroxide solution for 5 min, washed with PBS, and blocked with a horse serum solution (Vectastain Elite ABC, Vector Laboratories, Burlingame, CA, United States) for 10 min at room temperature (RT). After blocking, sections were washed in PBS and incubated with an antibody against GFAP 1:100 (Sigma) or BMP8 1:200 (Cell signaling technology) for 1 h at RT. After washing with PBS, sections were incubated with a biotinylated secondary antibody for 10 min. Then, sections were washed with PBS and incubated with the amplifier solution from the Vectastain Elite kit for 10 min at RT. The reaction was visualized using ImmunoDetector DAB (SB Bio Inc., Santa Barbara, CA, United States).

For counterstaining, sections were stained with hematoxylin for 30 s, dehydrated in baths containing ascending concentrations of alcohol and xylene, and mounted with synthetic resin.

For the IR analysis, for each mouse, three digital images were randomly selected at 40X magnification in the areas of interest (hippocampus and frontal cortex) (*n* = 5 mice/condition). The images were taken using a digital camera attached to a light microscope (Micrometrics, MilesCo Scientific, Princeton, MN, United States) connected to a camera operated by commercial software (Micrometrics SE Premium). The determination of the area of the immunolabeling was calculated delimiting the IR areas using the ImageJ software (National Institute of Health).

For these analyses, GFAP + astrocytes were selected randomly from the frontal cortex and the CA1, CA2, and dentate gyrus regions of the hippocampus and quantified. Using a digital camera on an inverted microscope, black and white images of GFAP + astrocytes were obtained and processed with Image J software. Using a 20X objective, cells were chosen randomly in the same area selected for immunostaining, and the binary overlay of a cell was created by thresholding. For all images a threshold value was established at the level at which the binary overlay entirely enclosed the cell body and projections. All pixels above the threshold value were considered as belonging to the cell images. Finally, the binary silhouette of the whole cell was reduced to its one-pixel outline for estimation of the fractal dimensions with the FracLac 2.5 ImageJ plug-in Karperien and Jelinek (2015).

### Statistical Analysis

All values were expressed as a mean ± standard error of the mean. The behavioral differences between treatment groups were assessed by one-way or two-way analysis of variance (ANOVA) with *post hoc* Tukey analysis. *P* < 0,05 was considered statistically significant. All statistical analyses were performed with the software GraphPad Prism 8 (GraphPad Software Inc., San Diego, CA, United States).

## Results

### Cotinine Infused in the mPFC Enhanced Fear Extinction in the Conditioned Mice

**FIGURE 2:**
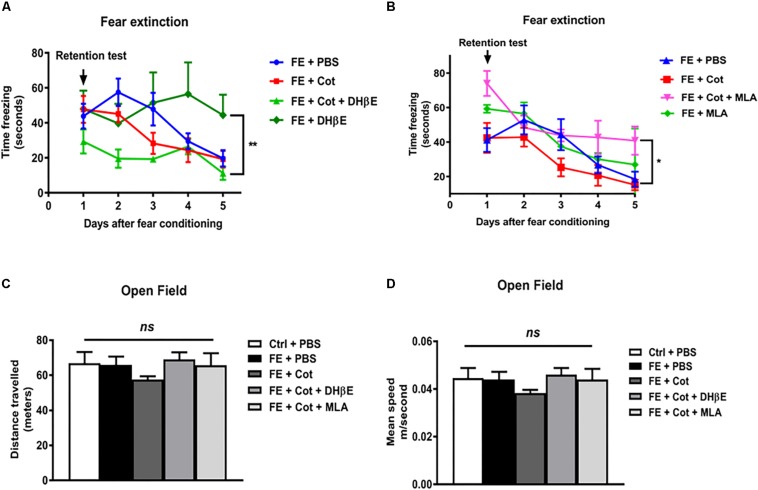
Effect of cotinine infused into the prefrontal cortex on fear conditioning and extinction. Male mice, treated as described in Materials and Methods section, were subjected to fear conditioning (FC) **(A)** and fear extinction **(B)** for five consecutive days in absence of presence of cotinine (Cot) treatment alone or plus MLA (α7 nAChR antagonist) or DHβE (α4β2 nAChRs antagonist). Locomotor activity was also tested measuring distance travelled **(C)**, and speed **(D)** in the open field test. Statistical significance was evaluated using one-way ANOVA with Tukey post-test. ns, not significant, ** highly significant *P* < 0.01.

To determine whether the infusion of cotinine into the mPFC could alter FE, cotinine was infused into this region 1 week prior to FC and FE. In the extinction trials, all PBS-treated mice showed a decrease in freezing behavior with the time that reached a steady state decrease by day 5. A repeated measure ANOVA throughout the 5 days of extinction revealed a significant difference induced by treatments [*F*(3,23) = 7.231, *P* = 0.03] and days [*F*(4, 92) = 15.22, *P* < 0.0001) on the freezing behavior. *Post hoc* Tukey’s multiple comparison test revealed that the infusion of cotinine alone did not change the acquisition or recall of the fear memory. In the retention test, cotinine-treated conditioned mice, showed a similar freezing reaction than PBS-treated conditioned mice (*P* = 0.47). In the extinction trials, mice treated with cotinine plus DHβE showed a trend of reduced retention of fear memory when compared to mice treated with cotinine alone but the difference did not reach statistical significance respect to conditioned mice treated with cotinine alone. Mice treated with DHβE alone induced a significant decrease in FE when compared to DHβE plus cotinine (Figure 2A, *P* < 0.05). Conversely, mice treated with cotinine plus MLA showed a significant impairment in fear extinction when compared with PBS-treated and cotinine-treated fear conditioned mice (*P* < 0.05) (Figure 2B). The enhancement of extinction in cotinine-treated mice was lost in mice co-treated with Cotinine plus MLA. The reduction in FE was even more pronounced in the mice treated with MLA alone than mice treated with cotinine plus MLA (Figure 2B).

### Effect of Intracortical Cotinine on Locomotor Activity in the Open Field Test of Conditioned Mice

**FIGURE 3:**
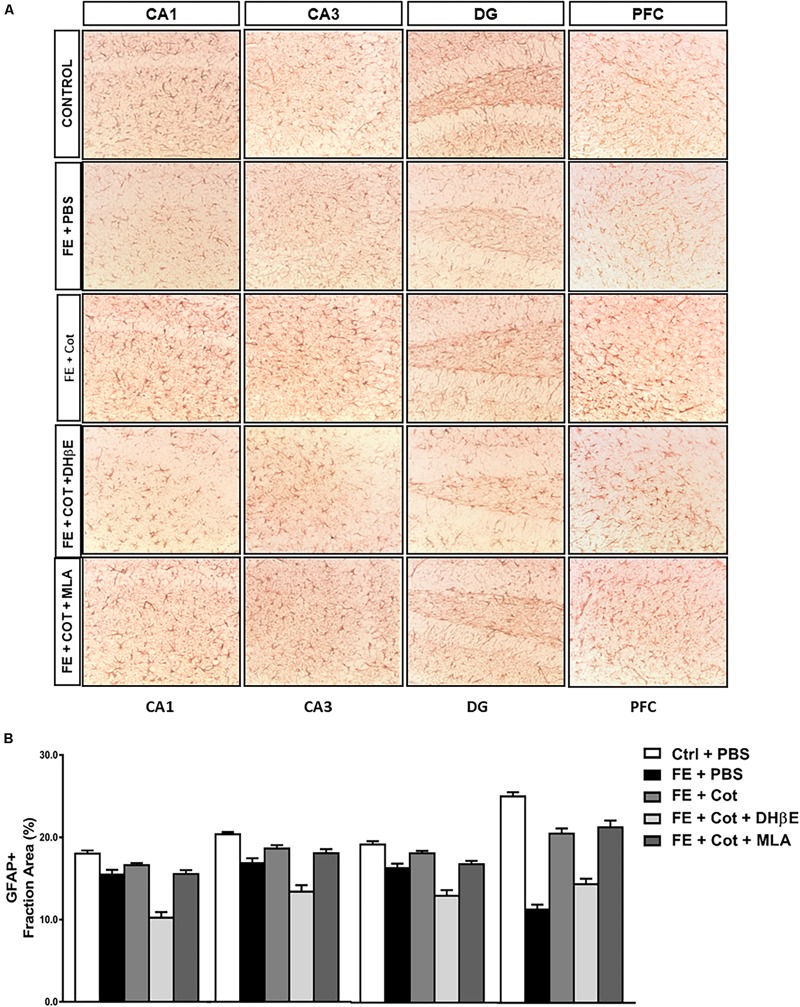
Cotinine prevented astrocyte loss in the prefrontal cortex and hippocampal formation of conditioned mice. After fear extinction (FE), mice (*n* = 5–8 mice/group) infused with cotinine (Cot) or Cot plus MLA but not with Cot + DHb E, showed higher GFAP immunoreactivity (IR) in the hippocampus regions [CA1, CA3 and the dentate gyrus (DG)], and medial prefrontal cortex (mPFC) of mice by an α4β2 nAChR-dependent mechanism. In the dentate gyrus (DG) also a significant effect of the inhibition of α7nAChR antagonistwas also observed. **(A)** The pictures despict the pictograms of representative immunohistochemical analysis of brain regions analyzed using a mouse anti-GFAP antibody; **(B)**, Plots representing the area of IR in the brain tissues showed in panel **A**. Statistical significance was evaluated using one-way ANOVA with Tukey posttest. ns, not significant, *** highly significant *P* < 0.001.

To determine the changes in locomotor activity induced by the infusion of drugs using stereotaxic techniques, mice were tested using the OF test for 25 min. The results did not show significant differences between treatment groups in distance travelled [*F*(4,26) = 0.981, *p* > 0.05; Figure 3A] or speed [*F*(4,26) = 0.9919, *P* > 0.05] (Figures2C,D).

### Effect of Cotinine Infused in the Medial Prefrontal Cortex on GFAP + Astrocytes Survival in the Brain of Conditioned Mice

Results demonstrate significant differences in GFAP + IR between treatment groups in the different regions of the HF, CA1 [F(4,60) = 48.68, *P* < 0.0001]; CA3 [*F*(4,60) = 33.62, *P* < 0.0001]; DG [*F*(4,61) = 43.23, *P* < 0.0001] (Figure 3A), and the PFC [*F*(4,65) = 75.46, *P* < 0.0001] (Figure 3B). Tukey’s *post hoc* analyses revealed a significant reduction of GFAP IR in the conditioned mice when compared to non-stressed mice in the CA1 (*P* < 0.0001), CA3 (*P* < 0.0001), and DG (*P* < 0.0001) of the HF, and the PFC (*P* < 0.0001) (Figure 3B).

The infusion of cotinine in the mPFC of the conditioned mice prevented the loss of astrocytes showing a significantly higher GFAP IR in the CA3 (*P* < 0.05), DG (*P* < 0.005), and PFC (*P* < 0.0001) than the one present in the PBS-treated conditioned mice (Figure 3B). The simultaneous treatment of the conditioned mice with cotinine plus MLA in the PFC did not prevent the effect of cotinine in enhancing GFAP IR, however, the infusion of DHβE prevented the increase of GFAP IR induced by cotinine in the conditioned mice that showed GFAP IR not significantly different from the PBS-treated conditioned mice (*P* < 0.05) and significantly different from the inhibitor alone (*P* < 0.001).

### Cotinine Downregulated BMP8 Expression in the Prefrontal Cortex of the Conditioned Mice

**FIGURE 4:**
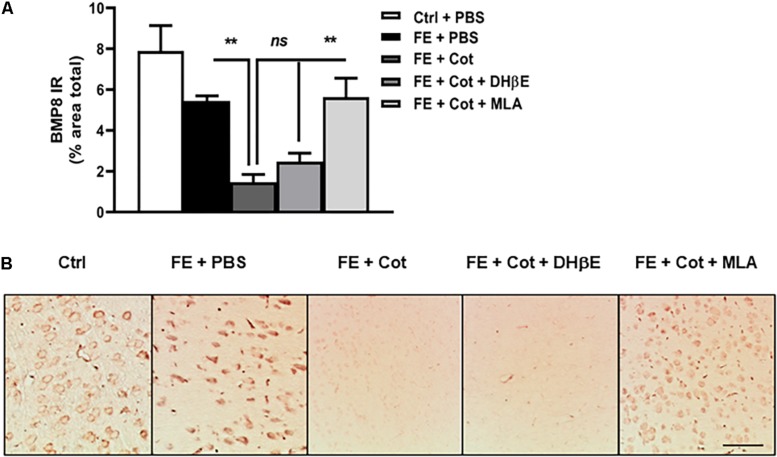
Cotinine decreased BMP8 expression in the prefrontal cortex of conditioned mice. Cotinine (Cot) almost suppressed the expression of BMP8 in the prefrontal cortex of mice as detected after fear fear extinction (FE). Cot or Cot plus DHβE-infused mice (*n* = 5–8/group) showed significantly lower levels of BMP8 than mice infused with PBS, or Cot plus MLA. **(A)** The plot represents BMP8 immunoreactivity (IR) levels after treatments **(B)**. The pictures depict the BMP8 IR in the medial prefrontal cortex (mPFC) as measured after treatments. Cotinine inhibited BMP8 expression in the mPFC of mice by a α7nAChRs-dependent mechanism. Statistical significance was evaluated using one-way ANOVA with Tukey posttest. ns, not significant, ** highly significant *P* < 0.01. ES, exposed to stress (FC).

The analysis of the protein expression of BMP8 in the PFC revealed a significant change in the expression of BMP8 between treatment groups [One-way ANOVA, *F*(4,56) = 12,33, *P* < 0.0001]. *Post hoc* analysis showed that after FE, the total BMP8 IR was not different between control non-conditioned mice and the conditioned mice after FE (ns, *P* = 0.651). However, after FE the cotinine-treated mice showed significantly lower levels of BMP8 IR than PBS-treated mice in the PFC (75% decrease, ^∗∗^*P* < 0.005) (Figures 4A,B). Conditioned mice treated with cotinine plus MLA showed BMP8 IR levels in the mPFC were not significantly different from conditioned mice infused with cotinine alone, or non-stressed mice (*P* > 0.05) (Figures 4A,B). The antagonism of the α4β2 receptors with DHβE in the mPFC did not prevent the downregulation of BMP8 induced by cotinine.

### Effect of Cotinine on BMP2 Levels in the Prefrontal Cortex of the Conditioned Mice

**FIGURE 5:**
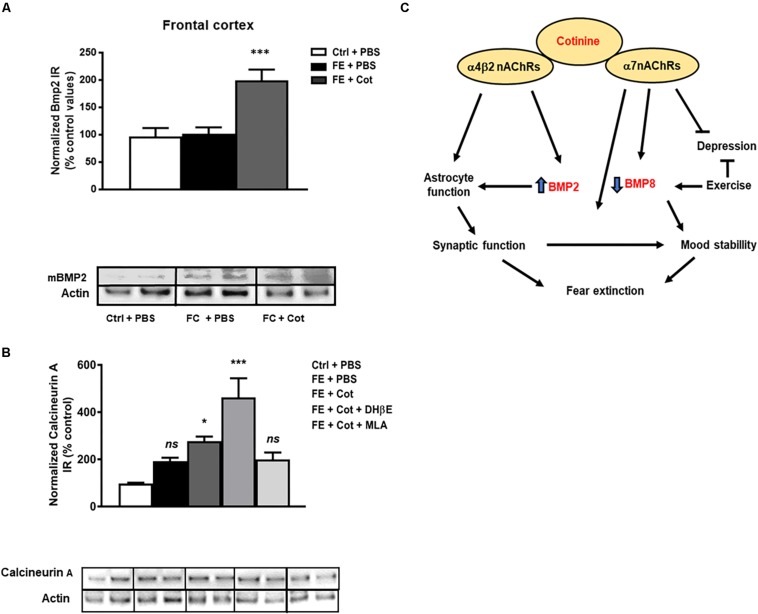
Cotinine increased BMP2 and calcineurin A expression in the prefrontal cortex of conditioned mice. Calcineurin A (CaA) immunoreactivity (IR) was higher in the prefrontal cortex (PFC) of cotinine-treated conditioned mice than PBS- treated conditioned mice or control non-shocked mice after fear extinction (*n* = 8–10/group). Cotinine-treated mice showed significantly higher levels of BMP2 than the vehicle-treated and non-shocked mice. BMP2 **(A)** and CaA **(B)** IRs are represented as the percentage of control non-stressed mice values and normalized to total actin levels. **(C)** The diagram summarizes the hypothetical signaling pathways involved in the positive effects of cotinine on PTSD pathology. ES, exposed to stress (FC); Veh, vehicle; ns, not significant; Statistical significance was evaluated using one-way ANOVA with Tukey post-test. *, Significant *P* < 0.05. **, highly significant (*P* < 0.01). ***, highly significant *P* < 0.001.

The results demonstrate a significant difference in the expression of the mature form of BMP2 (mBMP2) between treatment groups [One-way ANOVA, *F*(2,15) = 12.69, *P* < 0.001). *Post hoc* analysis showed that cotinine-treated conditioned mice showed significantly higher levels of mBMP2 IR than the vehicle-treated conditioned and non-conditioned mice in the PFC (*P* < 0.001) (Figure 5A). The evaluation of the effect of the antagonism of the nAChRs on cotinine’s actions revealed that neither MLA or DHβE prevented the increase induced by cotinine on BMP2 level in the mPFC (*P* > 0.05) (Figure 5A and Supplementary Figures 1a–c).

### Effect of Cotinine on Calcineurin a Level in the Prefrontal Cortex of Conditioned Mice

It has been shown that Calcineurin A (CaA) is involved in FE and cotinine, alike other antidepressants, increases its expression in the HIP (Alvarez-Ricartes et al., 2018b). The results showed a significant difference in CaA expression between treatment groups [One-way ANOVA, *F*(4,32) = 10.44, *P* < 0.0001]. The *post hoc* analysis demonstrated that after FE, the cotinine-treated conditioned mice showed a higher level of CaA than the conditioned mice treated with PBS in the PFC (77% increase *P* < 0.05). The evaluation of the effect of the antagonism of the nAChRs on cotinine’s actions revealed that MLA prevented the increase induced by cotinine on CaA level in the mPFC (*P* < 0.001) (Figure 5B and Supplementary Figures 2a–c).

## Discussion

The understanding of the molecular mechanisms involved in cotinine’s action is fundamental to discover new therapeutic targets to promote FE in affected individuals. In here, it was discovered that cotinine infusion in the mPFC enhanced contextual FE and prevented astrocyte loss by mechanisms involving the α7 and α4β2 nAChRs, respectively. In addition, it was found that cotinine increased BMP2 and decreased BMP8 expression in the mPFC as evaluated after FE. The evidence also suggests that cotinine acting on the nAChRs is required to enhance FE by modulating the activity of the mPFC and its connectivity with other regions of the fear loop such as the AMY and the HIP.

The inhibition of α4β2 nAChR with DHβE did not have the same effect that cotinine plus DHβE, indicating that the changes in behavior induced by the concommitant inhibition of the α4β2 receptors during cotinine treatments were not the result of the effect of the inhibitor itself, but the result of the inhibition of cotinine acting on this nicotinic receptor. We propose that DHβE alone freeze the receptor in its desensitized state but cotinine inhibits this effect by altering the receptor protein conformation. Previous studies showed that nicotine ameliorated fear conditioning that has been blocked by glutamate receptor inhibitors such as MK801 and that infusion of DHβE into the HIP negatively affected the positive effects of nicotine on contextual FC (Andre et al., 2008).

It has been proposed that different processes in the mPFC are involved in the acquisition and recall of fear learning. Because several neurons containing nAChRs project from the mPFC to the HIP and AMY (Pitkanen et al., 2000), the nAChR’s antagonists may interrupt the connectivity between the mPFC and these regions also involved in FE. The mPFC projects to several cortical and subcortical structures, including the AMY, insular cortex, lateral hypothalamus, periaqueductal gray area and rostral ventrolateral medulla (Vertes, 2004, 2006; Hoover and Vertes, 2007). Previous studies revealed that lesions of the infralimbic cortex (IL) in the mPFC resulted in a significant spontaneous recovery of conditioned fear responses after extinction in rats (Quirk et al., 2000). Changes in IL synaptic activity occurs during the acquisition (Muigg et al., 2008) and expression of extinction (Orsini et al., 2011, 2013; Orsini and Maren, 2012). Recent findings indicate that the IL target the basolateral AMY, which in turn recruits the intercalated cells to suppress fear (Strobel et al., 2015).

In addition to the mPFC, the HIP is important for the retrieval of FE memory associated with context (Maren et al., 2013). The modulation of the fear renewal with the expression of the context-dependent fear memories after extinction, involves enhanced activity of hippocampal neurons proyecting to the AMY and mPFC (Knapska and Maren, 2009; Knapska et al., 2012). In agreement with this evidence, it has been shown that cotinine by acting mainly in the PFC can positively influence astrocyte survival in the HIP.

Previous studies found that infusion of MLA or DHβE into the mPFC enhanced contextual FC. The data suggest that most likely α4β2 nAChRs mediate the enhancement of contextual fear conditioning by nicotine (Raybuck et al., 2008; Raybuck and Gould, 2009, 2010). In here, it was that retention of fear memory was not affected by infusion of cotinine into the mPFC, however, cotinine plus the selective inhibitor of the α7nAChR induced higher retention of the fear memory. Conversely, the infusion of cotinine plus DHβE inhibited the fear response to the context in the retention test.

Cotinine could prevent the enhancing of FC induced by DHβE by acting on the α4β2 nAChRs in the mPFC, and preventing the conformational changes induced by DHβE resulting in closed or resting states. Future studies using siRNA technology and additional antagonists would be required to discard the unspecific effects of these antagonists on other cell factors. Mistaken effects are unlikely considering the pharmacological profile of these nicotinic antagonists is well known and the doses used are in the range of doses previously investigated in the same strain of mice at the same ages. More inteerstingly a previous study has shown that the positive modulation of the α7nAChR in astrocytes inhibits the inflammatory response induced by LPS (Patel et al., 2017). Similarly, we found the effects of cotinine on astrocyte structure and numbers.

Astrocytes represent more than 60% of the cells in the brain, and have many functions in the CNS. These functions include modulating the blood-brain barrier, supporting neuronal plasticity and survival, providing neurons with energy molecules and trophic factors and removing glutamate from the extracellular space (Ashton et al., 2012; Honsek et al., 2012; Navarrete et al., 2012).

Astrocytes function is affected in PTSD with stress significantly decreasing the survival of astrocytes (Imbe et al., 2012; Perez-Urrutia et al., 2017). A substantial reduction in the number of astrocytes in the HIP and PFC of fear conditioned and restrained rodents have been found. However, IN cotinine (Alvarez-Ricartes et al., 2018b) and oral cotinine (Perez-Urrutia et al., 2017) prevented astrocyte loss induced by stress in the HIP and PFC of mice. The protective effect of cotinine toward astrocytes in these regions during stress was prevented by the antagonism of the α4β2 nAChRs with DHβE, but not by MLA, with the exception of the DG that also showed a minor effect of cotinine induced by MLA. This evidence suggests that the effect of cotinine on astrocyte survival is mainly mediated by the α4β2 nAChRs. The involvement of other receptors such as the α7b2 receptors mediating the effects of cotinine cannot be discarded. GABAergic interneurons in the CA1 subregion of the hippocampus express functional α7β2 nAChRs, which show pharmacological sensitivity to DHβE (Liu et al., 2012).

In addition, the facilitation of FE by cotinine was accompanied by a decrease of BMP8 and the increase of mBMP2 expression in the FC. Like other members of the TGFb superfamily, BMPs are synthesized as pre-proproteins synthesized at the rough endoplasmic reticulum (ER). In the secretory pathway, a BMP proprotein is cleaved on the C-terminal side by convertases, to release the C-terminal peptide. The C-terminal peptide homodimerizes by disulfide bonding to form the mature secreted BMP (Sopory et al., 2006). Active BMP dimers result in the activation of the SMAD proteins. It has been shown that proBMP-2 binds to BMP receptor IA with a similar affinity to its mature form (Hauburger et al., 2009), however, it induces a more delayed effect on gene expression when compared to the mature form of the peptide (von Einem et al., 2011).

Basolateral amygdalas also activate non-Smad signaling pathways, including the mitogen-activated kinases (MAPKs) p38 kinase, Erks, and c-Jun kinase (cJNK) as well as the phosphoinositide 3-kinase (PI3K)/Akt pathway (Mueller and Nickel, 2012). Interestingly, cotinine enhanced FE and activated ERK, Akt and CaA in the hippocampus of male mice (Grizzell et al., 2014; Alvarez-Ricartes et al., 2018b).

More importantly, like its effect on FE, the cotinine-induced decrease in Bmp8 was dependent on the activity of the α7nAChRs, but not by the antagonism of the α4β2 nAChRs.

The actual evidence is the first time that a connection between the effect of cotinine on astrocytes or behavior with the changes in BMPs expression. Previous evidence showing that BMPIIR deletion promoted neurogenesis and reduced anxiety behavior in rodents, supports the view that BMPs are involved in the regulation of mood and cognitive abilities in mice during stress (McBrayer et al., 2015).

The promoter region of the gene that codifies for BMP8 contains four GC response elements (GREs). In fact, dexamethazone upregulates BMP8 expression suggesting a role mediating GC actions. In the PFC of non-stressed mice, the IR for BMP8 was detected surrounding membranes, but the pattern of distribution looks different in the PFC of conditioned mice. Previous evidence suggests that inhibition of BMP8 mediates the positive effects of exercise on synaptic plasticity and learning (Gobeske et al., 2009).

BMP2 is one of the most potent osteo-inductive BMPs and one that has been approved by the American Food and Drug Administration for clinical use for spinal fusions and bone healing in humans (Govender et al., 2002). However, undesirable side effects limit its use (Benglis et al., 2008).

Basolateral amygdala signaling is active in adult neural stem cells and it is crucial to adult neurogenesis in the subependymal zone. Conditional deletion of Smad4 or infusion of Noggin, an extracellular antagonist of BMP, in adult neural stem cells suppressed neurogenesis and lead to increased differentiation in oligodendrocyte (Colak et al., 2008). In addition, a nuclear variant of Bmp2 (nBmp2) that is translated from an alternative start codon and has a nuclear localization signal overlapping the site of proteolytic cleavage (Felin et al., 2010).

It is intriguing that BMP8 immunoreactivity was detected around the plasma membrane in the non-conditioned mice. However, the pattern of distribution changed with stress. New investigations are required to assess the effect of stress on BMP8 translocation to other cell compartments in the brain.

The decrease in BMP8 and the increase of BMP2 involved the α7- and α4β2 nAChRs, respectively. These changes can be involved in the enhancement of FE, and the preservation of astrocytes function. It is known that BMP2 participates in astrogenesis and may also play a role a role in preventing astrocyte decrease induced by cotinine during stress conditions. In addition, the α7 receptor, which is positively modulated by cotinine, is present in the astrocytes and microglia, and its activation reduces neuroinflammation in several neurologial conditions (Shytle et al., 2004; Rehani et al., 2008; Foucault-Fruchard and Antier, 2017; Hoover, 2017). Thus an effect of cotinine on microglia is very likely. Consistent with this idea, it has been shown that cotinine inhibits the activation of monocytes. Pre-treatment of monocytes with cotinine changes the inflammatory response to Gram negative bacteria inhibiting the production of cytokines upregulated by NF-kappaB signaling such as TNF-a, interleukin (IL)-1b, IL-6, IL-12/IL-23 p40, while stimulating the expression of the anti-inflammatory IL-10. This anti-inflammatory effect was attained by acting on the monocytic α7 nAChR by a mechanism involving the PI3K/GSK-3beta pathway; but not NF-kB. Cotinine can prevent the activation of microglia acting on these receptors to reduce neuroinflammation and the consequent synaptic deficit and brain disconnection induced by stress. The modulation of the stress response by BMP proteins under conditions of stress, may also be controlled by mechanisms involving the nicotinic receptors and their downstream signaling factors. Thus, the investigation of the BMPs as new therapeutic targets for PTSD is guarantee.

## Conclusion

Based on this evidence, we conclude that cotinine favors astrocyte survival, and reduce the effect of stress on fear memory by acting on the α7-α4β2 nAChR. Also, it is possible to hypothesize that BMP2 and BMP8 proteins are involved in fear retention and extinction in subjects that suffered chronic or traumatic unscapable stress. Cotinine-induced inhibition of BMP8 and stimulation of BMP2 expression may facilitate extinction (Figure 5C). On the other hand, the positive modulation of the α7nAChR may protect astrocytes, reduce inflammatory responses and support cognition and mood stability. Cotinine may induce BMP2 expression by activating transcription factors downstream of ERK such as Sp1 (Kawamata and Shimohama, 2011), and this increase may promote synaptic plasticity and astrogenesis in the mPFC.

## Author’s Note

This material is the result of work supported with resources and the use of facilities at the Bay Pines VA Healthcare System FL, United States and the University San Sebastian, Chile. The contents do not represent the views of the Department of Veterans Affairs or the United States Government.

## Data Availability Statement

The datasets generated for this study are available on request to the corresponding author.

## Ethics Statement

The animal study was reviewed and approved by the Ethical Committee of Universidad san Sebastian.

## Author Contributions

All authors participated in all aspects of the manuscript and revised the submitted version.

## Conflict of Interest

VE was the inventor of two patents for the use of cotinine to facilitate fear memory extinction United States 20100104504 (United States Department of Veterans Affairs, University of South Florida). The remaining authors declare that the research was conducted in the absence of any commercial or financial relationships that could be construed as a potential conflict of interest.

## Abbreviations

AMY: Amygdala
ANOVA: analysis of variance
BLA: basolateral amygdala
BMPs: the bone morphogenetic proteins
CS: Conditioned Stimulus
DH β E: dihydro-beta-erythroidine
FE: fear memory extinction
GFAP: glial fibrillary acidic protein
HPA: hypothalamus-pituitary-adrenal
IN: intranasal
ITC: intercalated cells
MLA: methylcaconitine
mPFC: medial prefrontal cortex
MRI: magnetic resonance imaging
nAChRs: nicotinic acetylcholine receptors
OF: open field
PI3K: phosphoinositide-3 kinase
PTSD: posttraumatic stress disorder
TBS: Tris-buffered saline
TBST: TBS with 0.1% Tween 20
TGF: transforming growth factor.

## References

[B1] Alvarez-Ricartes N. Oliveros-Matus P. Mendoza C. Perez-Urrutia N. Echeverria F. Iarkov A. (2018a). Correction to: intranasal cotinine plus krill oil facilitates fear extinction, decreases depressive-like behavior, and increases hippocampal calcineurin a levels in mice. *Mol. Neurobiol.* 55:7961. 10.1007/s12035-018-1095-8 29744810

[B2] Alvarez-Ricartes N. Oliveros-Matus P. Mendoza C. Perez-Urrutia N. Echeverria F. Iarkov A. (2018b). Intranasal cotinine plus krill oil facilitates fear extinction, decreases depressive-like behavior, and increases hippocampal calcineurin a levels in mice. *Mol. Neurobiol.* 55 7949–7960. 10.1007/s12035-018-0916-0 29488138

[B3] Andre J. M. Gulick D. Portugal G. S. Gould T. J. (2008). Nicotine withdrawal disrupts both foreground and background contextual fear conditioning but not pre-pulse inhibition of the acoustic startle response in C57BL/6 mice. *Behav. Brain Res.* 190 174–181. 10.1016/j.bbr.2008.02.018 18367257 PMC2409068

[B4] Ashton R. S. Conway A. Pangarkar C. Bergen J. Lim K. I. Shah P. (2012). Astrocytes regulate adult hippocampal neurogenesis through ephrin-B signaling. *Nat. Neurosci.* 15 1399–1406. 10.1038/nn.3212 22983209 PMC3458152

[B5] Benglis D. Wang M. Y. Levi A. D. (2008). A comprehensive review of the safety profile of bone morphogenetic protein in spine surgery. *Neurosurgery* 62(5 Suppl. 2), ONS423–ONS431. 10.1227/01.neu.0000326030.24220.d8 18596525

[B6] Besson M. Granon S. Mameli-Engvall M. Cloëz-Tayarani I. Maubourguet N. Cormier A. (2007). Long-term effects of chronic nicotine exposure on brain nicotinic receptors. *Proc. Natl. Acad. Sci. U.S.A.* 104 8155–8160. 10.1073/pnas.0702698104 17470777 PMC1859991

[B7] Colak D. Mori T. Brill M. S. Pfeifer A. Falk S. Deng C. (2008). Adult neurogenesis requires Smad4-mediated bone morphogenic protein signaling in stem cells. *J. Neurosci.* 28 434–446. 10.1523/JNEUROSCI.4374-07.2008 18184786 PMC6670509

[B8] Felin J. E. Mayo J. L. Loos T. J. Jensen J. D. Sperry D. K. Gaufin S. L. (2010). Nuclear variants of bone morphogenetic proteins. *BMC Cell Biol.* 11:20. 10.1186/1471-2121-11-20 20230640 PMC2850327

[B9] Foucault-Fruchard L. Antier D. (2017). Therapeutic potential of alpha7 nicotinic receptor agonists to regulate neuroinflammation in neurodegenerative diseases. *Neural Regen. Res.* 12 1418–1421. 10.4103/1673-5374.215244 29089979 PMC5649454

[B10] Gobeske K. T. Das S. Bonaguidi M. A. Weiss C. Radulovic J. Disterhoft J. F. (2009). BMP signaling mediates effects of exercise on hippocampal neurogenesis and cognition in mice. *PLoS One* 4:e7506. 10.1371/journal.pone.0007506 19841742 PMC2759555

[B11] Govender S. Csimma C. Genant H. K. Valentin-Opran A. Amit Y. Arbel R. (2002). Recombinant human bone morphogenetic protein-2 for treatment of open tibial fractures: a prospective, controlled, randomized study of four hundred and fifty patients. *J. Bone Joint Surg. Am.* 84 2123–2134. 10.2106/00004623-200212000-00001 12473698

[B12] Grizzell J. A. Iarkov A. Holmes R. Mori T. Echeverria V. (2014). Cotinine reduces depressive-like behavior, working memory deficits, and synaptic loss associated with chronic stress in mice. *Behav. Brain Res.* 268 55–65. 10.1016/j.bbr.2014.03.047 24713149

[B13] Hauburger A. von Einem S. Schwaerzer G. K. Buttstedt A. Zebisch M. Schräml M. (2009). The pro-form of BMP-2 interferes with BMP-2 signalling by competing with BMP-2 for IA receptor binding. *FEBS J.* 276 6386–6398. 10.1111/j.1742-4658.2009.07361.x 19804412

[B14] Honsek S. D. Walz C. Kafitz K. W. Rose C. R. (2012). Astrocyte calcium signals at Schaffer collateral to CA1 pyramidal cell synapses correlate with the number of activated synapses but not with synaptic strength. *Hippocampus* 22 29–42. 10.1002/hipo.20843 20882545

[B15] Hoover D. B. (2017). Cholinergic modulation of the immune system presents new approaches for treating inflammation. *Pharmacol. Ther.* 179 1–16. 10.1016/j.pharmthera.2017.05.002 28529069 PMC5651192

[B16] Hoover W. B. Vertes R. P. (2007). Anatomical analysis of afferent projections to the medial prefrontal cortex in the rat. *Brain Struct. Funct.* 212 149–179. 10.1007/s00429-007-0150-4 17717690

[B17] Imbe H. Kimura A. Donishi T. Kaneoke Y. (2012). Chronic restraint stress decreases glial fibrillary acidic protein and glutamate transporter in the periaqueductal gray matter. *Neuroscience* 223 209–218. 10.1016/j.neuroscience.2012.08.007 22890077

[B18] Imura T. Tane K. Toyoda N. Fushiki S. (2008). Endothelial cell-derived bone morphogenetic proteins regulate glial differentiation of cortical progenitors. *Eur. J. Neurosci.* 27 1596–1606. 10.1111/j.1460-9568.2008.06134.x 18380662

[B19] Karperien A. L. Jelinek H. F. (2015). Fractal, multifractal, and lacunarity analysis of microglia in tissue engineering. *Front. Bioeng. Biotechnol.* 3:51. 10.3389/fbioe.2015.00051 25927064 PMC4396415

[B20] Kawamata J. Shimohama S. (2011). Stimulating nicotinic receptors trigger multiple pathways attenuating cytotoxicity in models of Alzheimer’s and Parkinson’s diseases. *J. Alzheimers. Dis.* 24(Suppl. 2), 95–109. 10.3233/JAD-2011-110173 21403387

[B21] Kilkenny C. Browne W. J. Cuthill I. C. Emerson M. Altman D. G. (2010). Improving bioscience research reporting: the ARRIVE guidelines for reporting animal research. *PLoS Biol.* 8:e1000412. 10.1371/journal.pbio.1000412 20613859 PMC2893951

[B22] Knapska E. Macias M. Mikosz M. Nowak A. Owczarek D. Wawrzyniak M. (2012). Functional anatomy of neural circuits regulating fear and extinction. *Proc. Natl. Acad. Sci. U.S.A.* 109 17093–17098. 10.1073/pnas.1202087109 23027931 PMC3479515

[B23] Knapska E. Maren S. (2009). Reciprocal patterns of c-Fos expression in the medial prefrontal cortex and amygdala after extinction and renewal of conditioned fear. *Learn. Mem.* 16 486–493. 10.1101/lm.1463909 19633138 PMC2726014

[B24] Laurier L. G. Corrigall W. A. George S. R. (1994). Dopamine receptor density, sensitivity and mRNA levels are altered following self-administration of cocaine in the rat. *Brain Res.* 634 31–40. 10.1016/0006-8993(94)90255-0 8156390

[B25] Laviolette S. R. van der Kooy D. (2003). The motivational valence of nicotine in the rat ventral tegmental area is switched from rewarding to aversive following blockade of the alpha7-subunit-containing nicotinic acetylcholine receptor. *Psychopharmacology* 166 306–313. 10.1007/s00213-002-1317-6 12569428

[B26] Liu Q. Huang Y. Shen J. Steffensen S. Wu J. (2012). Functional α7β2 nicotinic acetylcholine receptors expressed in hippocampal interneurons exhibit high sensitivity to pathological level of amyloid β peptides. *BMC Neurosci.* 13:155. 10.1186/1471-2202-13-155 23272676 PMC3573893

[B27] Maren S. Phan K. L. Liberzon I. (2013). The contextual brain: implications for fear conditioning, extinction and psychopathology. *Nat. Rev. Neurosci.* 14 417–428. 10.1038/nrn3492 23635870 PMC5072129

[B28] McBrayer Z. L. Dimova J. Pisansky M. T. Sun M. Beppu H. Gewirtz J. C. (2015). Forebrain-specific loss of BMPRII in mice reduces anxiety and increases object exploration. *PLoS One* 10:e0139860. 10.1371/journal.pone.0139860 26444546 PMC4596878

[B29] Mendoza C. Barreto G. E. Iarkov A. Tarasov V. V. Aliev G. Echeverria V. (2018). Cotinine: a therapy for memory extinction in post-traumatic stress disorder. *Mol. Neurobiol.* 55 6700–6711. 10.1007/s12035-018-0869-3 29335846

[B30] Morikawa M. Koinuma D. Mizutani A. Kawasaki N. Holmborn K. Sundqvist A. (2016). BMP sustains embryonic stem cell self-renewal through distinct functions of different kruppel-like factors. *Stem Cell Rep.* 6 64–73. 10.1016/j.stemcr.2015.12.004 26771354 PMC4719190

[B31] Mueller T. D. Nickel J. (2012). Promiscuity and specificity in BMP receptor activation. *FEBS Lett.* 586 1846–1859. 10.1016/j.febslet.2012.02.043 22710174

[B32] Muigg P. Hetzenauer A. Hauer G. Hauschild M. Gaburro S. Frank E. (2008). Impaired extinction of learned fear in rats selectively bred for high anxiety–evidence of altered neuronal processing in prefrontal-amygdala pathways. *Eur. J. Neurosci.* 28 2299–2309. 10.1111/j.1460-9568.2008.06511.x 19019199 PMC2777258

[B33] Navarrete M. Perea G. Fernandez de Sevilla D. Gomez-Gonzalo M. Nunez A. Martin E. D. (2012). Astrocytes mediate *in vivo* cholinergic-induced synaptic plasticity. *PLoS Biol.* 10:e1001259. 10.1371/journal.pbio.1001259 22347811 PMC3279365

[B34] Orsini C. A. Kim J. H. Knapska E. Maren S. (2011). Hippocampal and prefrontal projections to the basal amygdala mediate contextual regulation of fear after extinction. *J. Neurosci.* 31 17269–17277. 10.1523/JNEUROSCI.4095-11.2011 22114293 PMC3241946

[B35] Orsini C. A. Maren S. (2012). Neural and cellular mechanisms of fear and extinction memory formation. *Neurosci. Biobehav. Rev.* 36 1773–1802. 10.1016/j.neubiorev.2011.12.014 22230704 PMC3345303

[B36] Orsini C. A. Yan C. Maren S. (2013). Ensemble coding of context-dependent fear memory in the amygdala. *Front. Behav. Neurosci.* 7:199. 10.3389/fnbeh.2013.00199 24379767 PMC3861741

[B37] Panagis G. Kastellakis A. Spyraki C. Nomikos G. (2000). Effects of methyllycaconitine (MLA), an alpha 7 nicotinic receptor antagonist, on nicotine- and cocaine-induced potentiation of brain stimulation reward. *Psychopharmacology* 149 388–396. 10.1007/s002130000384 10867966

[B38] Patel H. McIntire J. Ryan S. Dunah A. Loring R. (2017). Anti-inflammatory effects of astroglial alpha7 nicotinic acetylcholine receptors are mediated by inhibition of the NF-kappaB pathway and activation of the Nrf2 pathway. *J. Neuroinflamm.* 14:192. 10.1186/s12974-017-0967-6 28950908 PMC5615458

[B39] Paxinos G. Franklin K. B. J. (2001). *The Mouse Brain in Stereotaxic Coordinates.* San Diego, CA: Academic Press.

[B40] Perez-Urrutia N. Mendoza C. Alvarez-Ricartes N. Oliveros-Matus P. Echeverria F. Grizzell J. A. (2017). Intranasal cotinine improves memory, and reduces depressive-like behavior, and GFAP+ cells loss induced by restraint stress in mice. *Exp. Neurol.* 295 211–221. 10.1016/j.expneurol.2017.06.016 28625590

[B41] Pitkanen A. Pikkarainen M. Nurminen N. Ylinen A. (2000). Reciprocal connections between the amygdala and the hippocampal formation, perirhinal cortex, and postrhinal cortex in rat. A review. *Ann. N. Y. Acad. Sci.* 911 369–391. 10.1111/j.1749-6632.2000.tb06738.x 10911886

[B42] Quirk G. J. Russo G. K. Barron J. L. Lebron K. (2000). The role of ventromedial prefrontal cortex in the recovery of extinguished fear. *J. Neurosci.* 20 6225–6231. 10.1523/jneurosci.20-16-06225.2000 10934272 PMC6772571

[B43] Raybuck J. D. Gould T. J. (2009). Nicotine withdrawal-induced deficits in trace fear conditioning in C57BL/6 mice–a role for high-affinity beta2 subunit-containing nicotinic acetylcholine receptors. *Eur. J. Neurosci.* 29 377–387. 10.1111/j.1460-9568.2008.06580.x 19200240 PMC2746945

[B44] Raybuck J. D. Gould T. J. (2010). The role of nicotinic acetylcholine receptors in the medial prefrontal cortex and hippocampus in trace fear conditioning. *Neurobiol. Learn. Mem.* 94 353–363. 10.1016/j.nlm.2010.08.001 20727979 PMC2949463

[B45] Raybuck J. D. Portugal G. S. Lerman C. Gould T. J. (2008). Varenicline ameliorates nicotine withdrawal-induced learning deficits in C57BL/6 mice. *Behav. Neurosci.* 122 1166–1171. 10.1037/a0012601 18823172 PMC2683368

[B46] Rehani K. Scott D. A. Renaud D. Hamza H. Williams L. R. Wang H. (2008). Cotinine-induced convergence of the cholinergic and PI3 kinase-dependent anti-inflammatory pathways in innate immune cells. *Biochim. Biophys. Acta* 1783 375–382. 10.1016/j.bbamcr.2007.12.003 18178163

[B47] Rutt B. T. Oehlert M. E. Krieshok T. S. Lichtenberg J. W. (2018). Effectiveness of cognitive processing therapy and prolonged exposure in the department of veterans affairs. *Psychol. Rep.* 121 282–302. 10.1177/0033294117727746 28886664

[B48] Shaked M. Weissmuller K. Svoboda H. Hortschansky P. Nishino N. Wolfl S. (2008). Histone deacetylases control neurogenesis in embryonic brain by inhibition of BMP2/4 signaling. *PLoS One* 3:e2668. 10.1371/journal.pone.0002668 18628975 PMC2441862

[B49] Shytle R. D. Mori T. Townsend K. Vendrame M. Sun N. Zeng J. (2004). Cholinergic modulation of microglial activation by alpha 7 nicotinic receptors. *J. Neurochem.* 89 337–343. 10.1046/j.1471-4159.2004.02347.x 15056277

[B50] Sopory S. Nelsen S. M. Degnin C. Wong C. Christian J. L. (2006). Regulation of bone morphogenetic protein-4 activity by sequence elements within the prodomain. *J. Biol. Chem.* 281 34021–34031. 10.1074/jbc.m605330200 16966322

[B51] Steenkamp M. M. Litz B. T. Hoge C. W. Marmar C. R. (2015). Psychotherapy for military-related PTSD: a review of randomized clinical trials. *JAMA* 314 489–500. 10.1001/jama.2015.8370 26241600

[B52] Strobel C. Marek R. Gooch H. M. Sullivan R. K. P. Sah P. (2015). Prefrontal and auditory input to intercalated neurons of the amygdala. *Cell Rep.* 10 1435–1442. 10.1016/j.celrep.2015.02.008 25753409

[B53] Terry A. V. Jr. Callahan P. M. Bertrand D. (2015). R-(+) and S-(-) isomers of cotinine augment cholinergic responses *in vitro* and *in vivo*. *J. Pharmacol. Exp. Ther.* 352 405–418. 10.1124/jpet.114.219881 25503389 PMC4293431

[B54] Vertes R. P. (2004). Differential projections of the infralimbic and prelimbic cortex in the rat. *Synapse* 51 32–58. 10.1002/syn.10279 14579424

[B55] Vertes R. P. (2006). Interactions among the medial prefrontal cortex, hippocampus and midline thalamus in emotional and cognitive processing in the rat. *Neuroscience* 142 1–20. 10.1016/j.neuroscience.2006.06.027 16887277

[B56] von Einem S. Erler S. Bigl K. Frerich B. Schwarz E. (2011). The pro-form of BMP-2 exhibits a delayed and reduced activity when compared to mature BMP-2. *Growth Fact.* 29 63–71. 10.3109/08977194.2011.561798 21391795

[B57] Walsh R. N. Cummins R. A. (1976). The Open-Field Test: a critical review. *Psychol. Bull.* 83 482–504. 10.1037/0033-2909.83.3.482 17582919

[B58] Wessa M. Flor H. (2007). Failure of extinction of fear responses in posttraumatic stress disorder: evidence from second-order conditioning. *Am. J. Psychiatry* 164 1684–1692. 10.1176/appi.ajp.2007.07030525 17974933

[B59] Yeomans J. Baptista M. (1997). Both nicotinic and muscarinic receptors in ventral tegmental area contribute to brain-stimulation reward. *Pharmacol. Biochem. Behav.* 57 915–921. 10.1016/s0091-3057(96)00467-4 9259024

[B60] Zeitlin R. Patel S. Solomon R. Tran J. Weeber E. J. Echeverria V. (2012). Cotinine enhances the extinction of contextual fear memory and reduces anxiety after fear conditioning. *Behav. Brain Res.* 228 284–293. 10.1016/j.bbr.2011.11.023 22137886

