# Supplementary figures and images for: Cotinine Enhances Fear Extinction and Astrocyte Survival by Mechanisms Involving the Nicotinic Acetylcholine Receptors Signaling
Source: Front Pharmacol. 2020 Apr 2;11:303. doi: 10.3389/fphar.2020.00303 (PMC7142247; doi:10.3389/fphar.2020.00303)

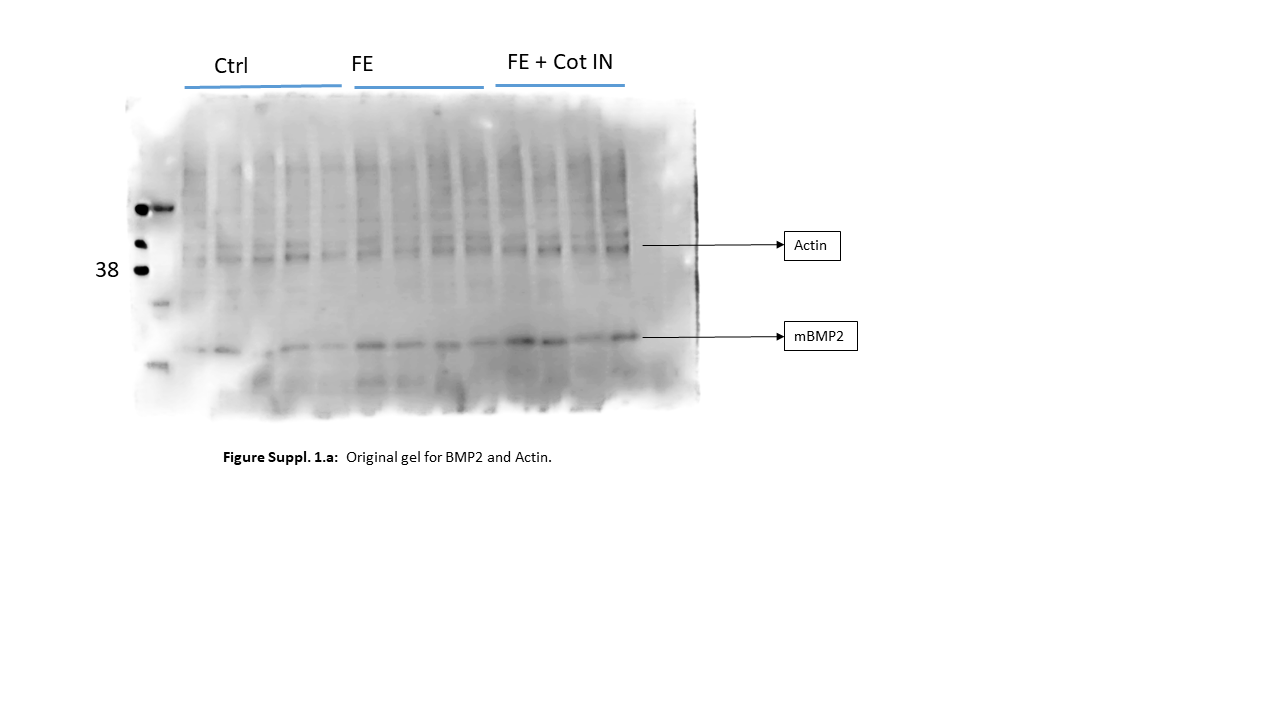

Supplement: Supplementary file 1 [file Data_Sheet_1.ZIP › Slide1.TIF]

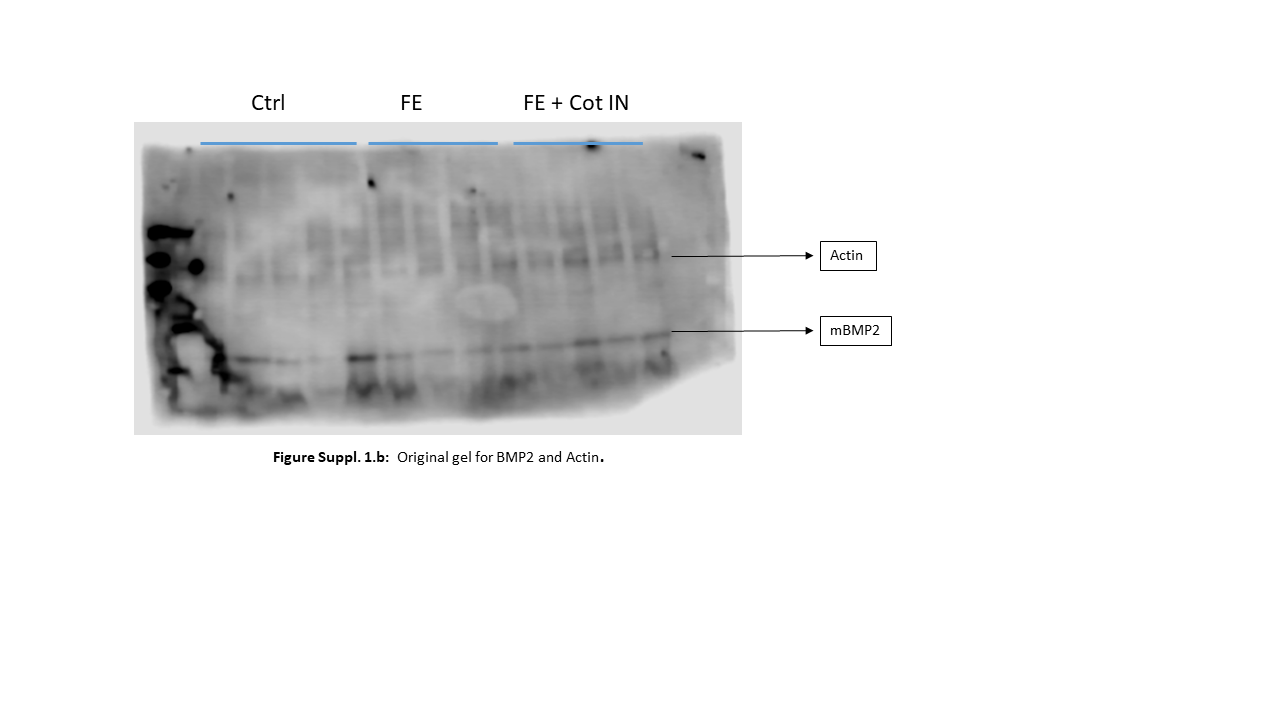

Supplement: Supplementary file 1 [file Data_Sheet_1.ZIP › Slide2.TIF]

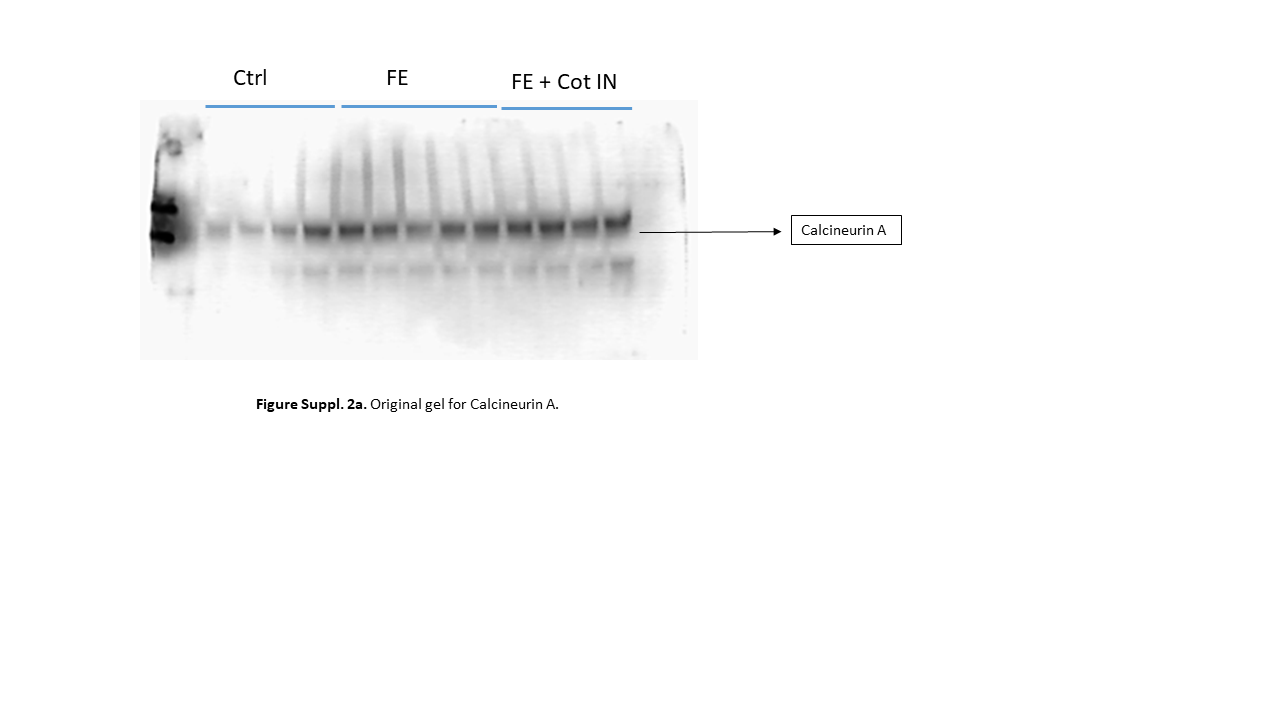

Supplement: Supplementary file 1 [file Data_Sheet_1.ZIP › Slide3.TIF]

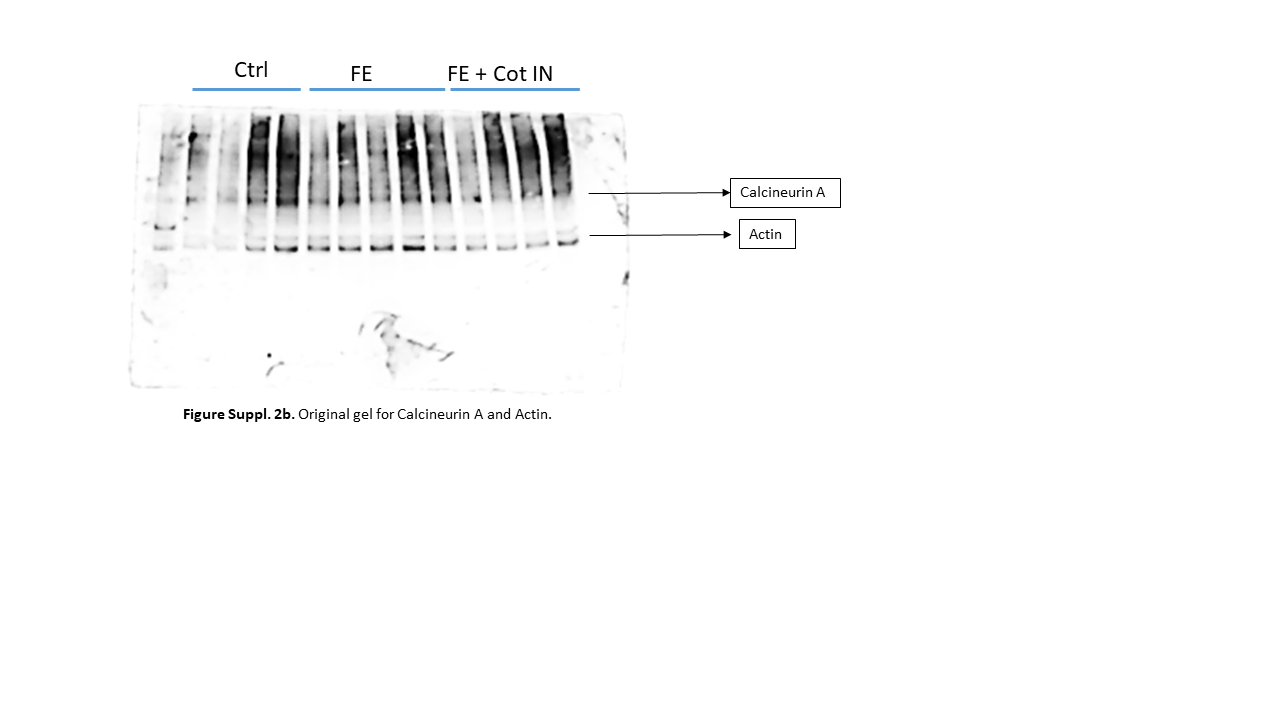

Supplement: Supplementary file 1 [file Data_Sheet_1.ZIP › Slide4.TIF]

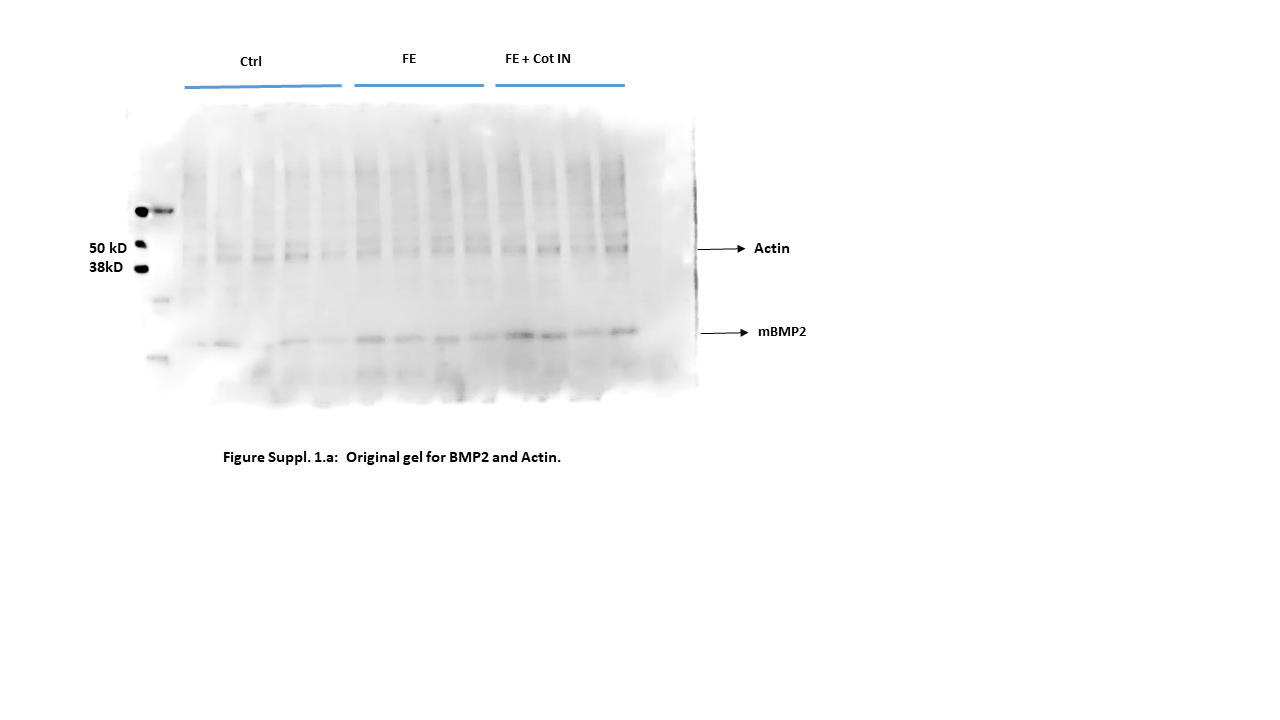

Supplement: Supplementary file 2 [file Data_Sheet_2.ZIP › SupplFig1a.TIF]

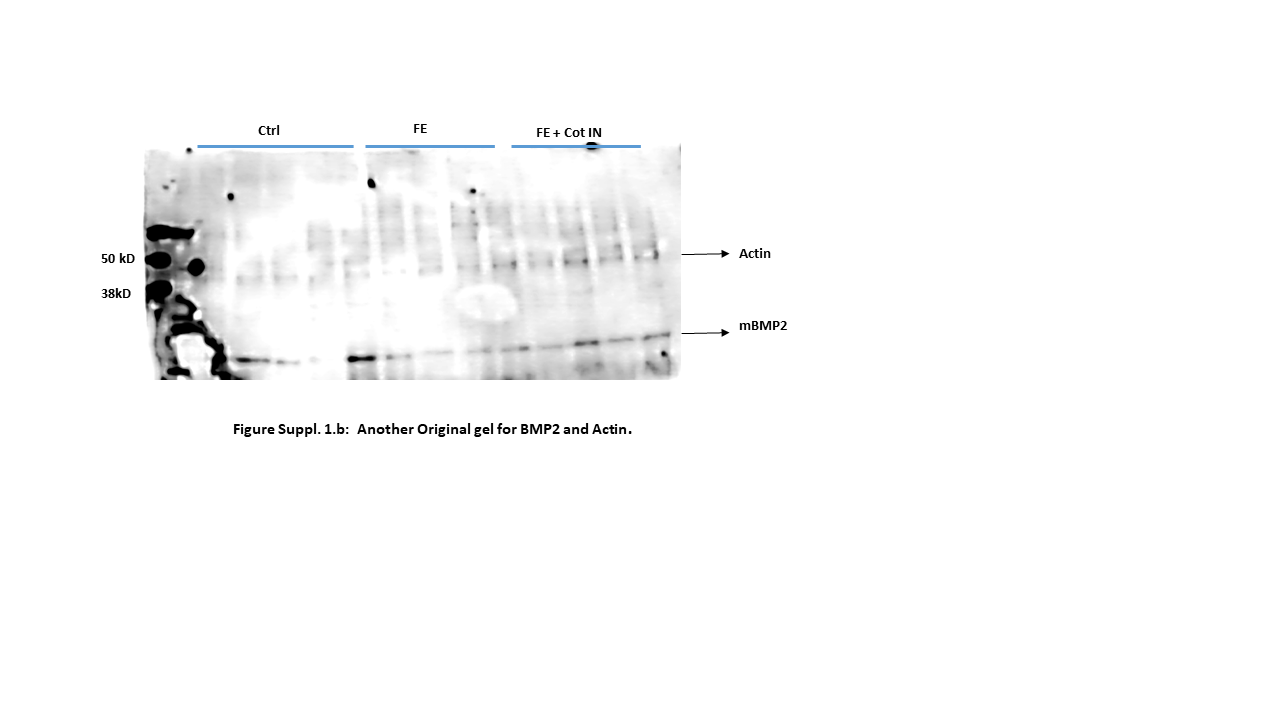

Supplement: Supplementary file 2 [file Data_Sheet_2.ZIP › SupplFig1b.TIF]

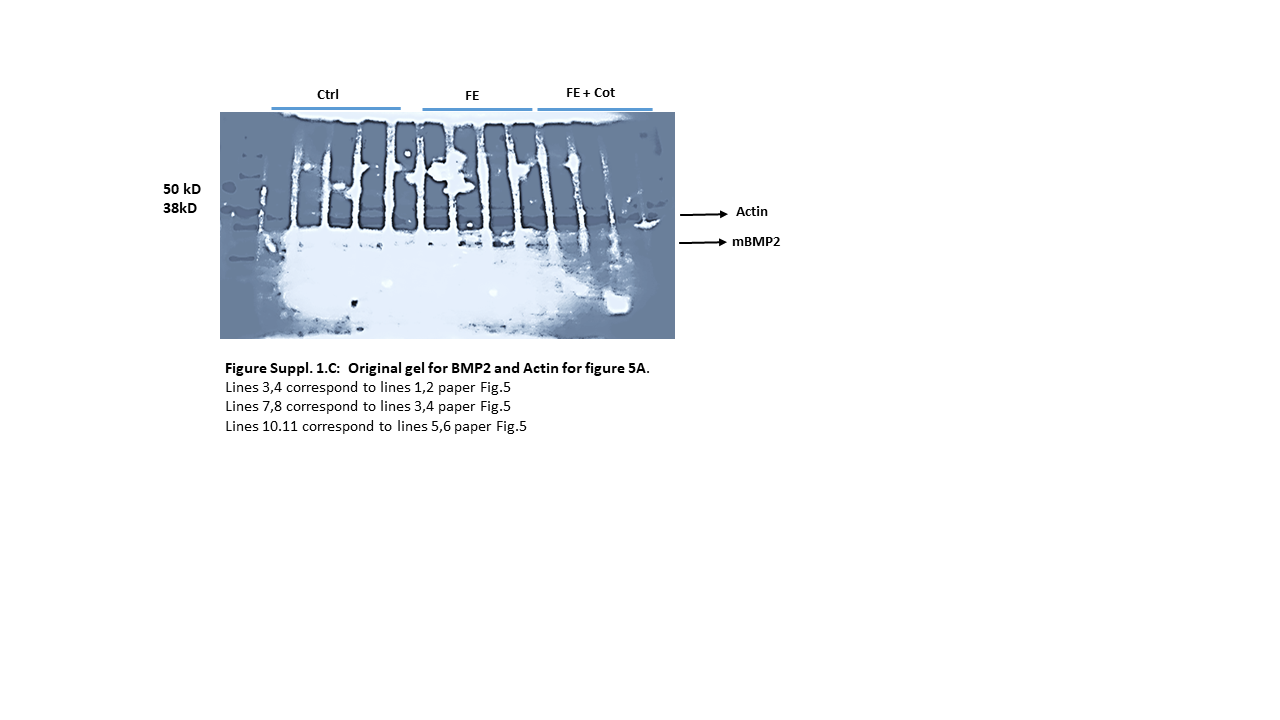

Supplement: Supplementary file 2 [file Data_Sheet_2.ZIP › SupplFig1c.TIF]

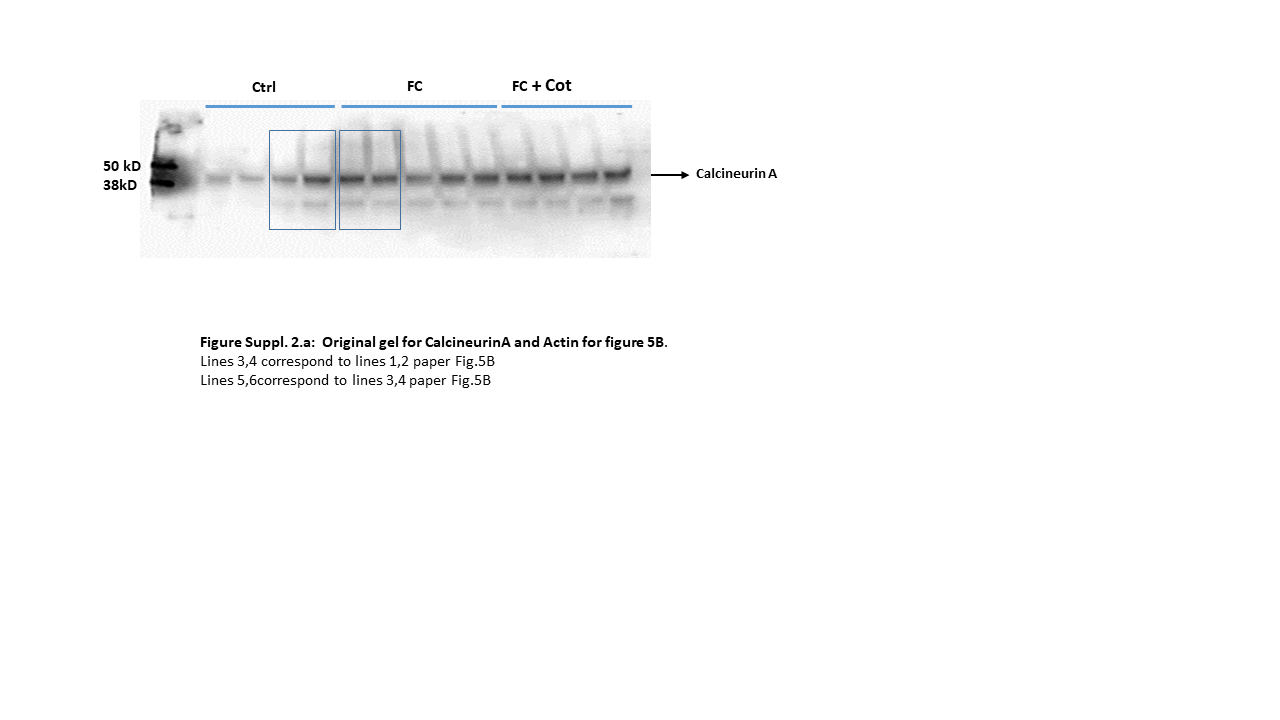

Supplement: Supplementary file 2 [file Data_Sheet_2.ZIP › SupplFig2a.TIF]

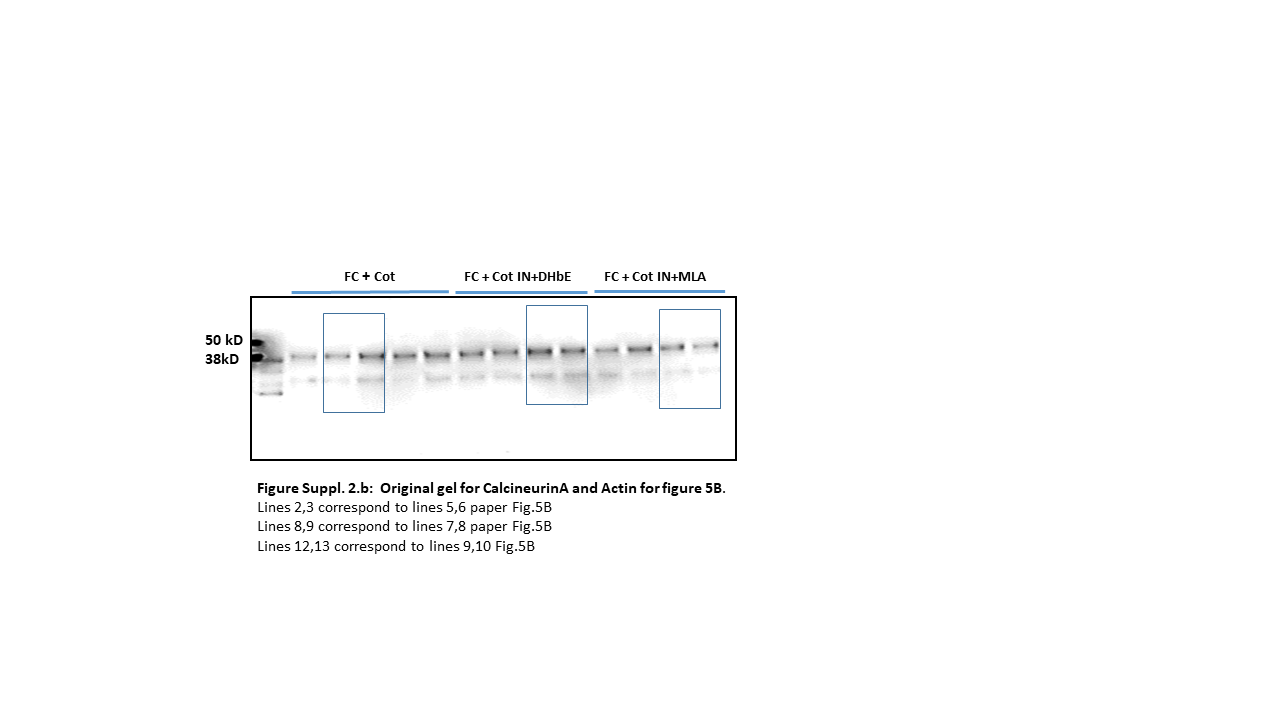

Supplement: Supplementary file 2 [file Data_Sheet_2.ZIP › SupplFig2b.TIF]

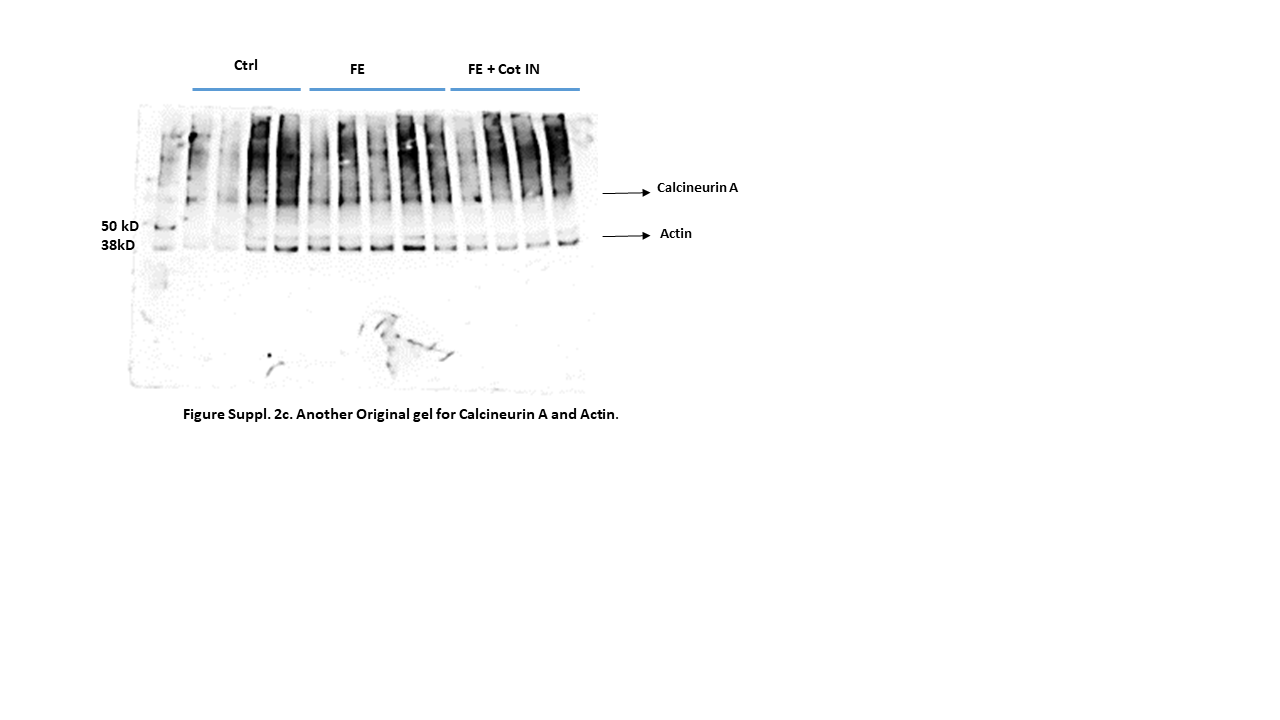

Supplement: Supplementary file 2 [file Data_Sheet_2.ZIP › SupplFig2c.TIF]
